# Enthalpy–entropy compensation: the role of solvation

**DOI:** 10.1007/s00249-016-1182-6

**Published:** 2016-10-28

**Authors:** Anatoliy I. Dragan, Christopher M. Read, Colyn Crane-Robinson

**Affiliations:** 1 Institute of High Technologies, Taras Shevchenko National University of Kyiv, 64, Volodymyrs’ka St., Kiev, 01601 Ukraine; 2 Institute of Molecular Biology and Genetics, NASU, 150, Zabolotnogo St., Kiev, 03680 Ukraine; 3 Biophysics Laboratories, School of Biology, University of Portsmouth, Portsmouth, PO1 2DT UK

**Keywords:** Enthalpy–entropy compensation (EEC), Solvation water, Hydration, Proteins, DNA

## Abstract

Structural modifications to interacting systems frequently lead to changes in both the enthalpy (heat) and entropy of the process that compensate each other, so that the Gibbs free energy is little changed: a major barrier to the development of lead compounds in drug discovery. The conventional explanation for such enthalpy–entropy compensation (EEC) is that tighter contacts lead to a more negative enthalpy but increased molecular constraints, i.e., a compensating conformational entropy reduction. Changes in solvation can also contribute to EEC but this contribution is infrequently discussed. We review long-established and recent cases of EEC and conclude that the large fluctuations in enthalpy and entropy observed are too great to be a result of only conformational changes and must result, to a considerable degree, from variations in the amounts of water immobilized or released on forming complexes. Two systems exhibiting EEC show a correlation between calorimetric entropies and local mobilities, interpreted to mean conformational control of the binding entropy/free energy. However, a substantial contribution from solvation gives the same effect, as a consequence of a structural link between the amount of bound water and the protein flexibility. Only by assuming substantial changes in solvation—an intrinsically compensatory process—can a more complete understanding of EEC be obtained. Faced with such large, and compensating, changes in the enthalpies and entropies of binding, the best approach to engineering elevated affinities must be through the addition of ionic links, as they generate increased entropy without affecting the enthalpy.

## Enthalpy–entropy compensation

Efforts to establish structure–activity relationships (SARs) and improve the affinity of drugs for target proteins typically involve thermodynamic measurements on a panel of modified forms of the lead compound, principally using isothermal titration calorimetry (ITC). It is frequently observed that whereas the Gibbs energy of binding, i.e., the binding constant, remains largely unchanged in consequence of the addition/subtraction of chemical groups, there are substantial variations in the component enthalpies and entropies. If ∆*G* remains the same, it follows that changes in ∆*H* and *T*∆*S* compensate one another. In fact, enthalpy-entropy compensation (EEC) is a widely observed phenomenon and is typically explained by assuming that if a molecular change in the ligand leads to more and/or tighter van der Waals contacts and H-bonds with the substrate (giving a more negative ∆*H*), this inevitably leads to reduced mobility/flexibility in either or both components of the interaction, i.e., a reduction in the overall conformational entropy, and that change compensates the enthalpy decrease. However, the amount of water hydrating the system can also change and if any of this water is tightly bound, its contribution to the enthalpy and entropy of binding will also be largely compensatory, as pointed out long ago (Lumry and Rajender 1970). On a simplified model, desorption of tightly bound water will have the thermodynamic characteristics of melting ice, i.e., large positive enthalpies and entropy factors that largely compensate each other. Although changes in water order are widely accepted as possible contributors, discussions of ligand binding thermodynamics, in particular the entropy changes, are normally restricted to comments on possible conformational alterations. However, in some cases, the evidence for the participation of water release or binding is very strong. The thesis put forward in this review is that a large proportion of the variation in the compensating enthalpy and entropy of macromolecular interactions comes from changes in the level of hydration, rather than conformational changes.

## Studies of ligand binding to macromolecules

In most published cases of enthalpy-entropy compensation, authors typically favor the ‘traditional’ conformational explanation and additionally acknowledge that changes in solvation may also play a role. We first illustrate EEC by giving four well-established examples, for the last of which ‘solvent reorganization’ is taken to be the sole explanation of the observed compensation.

1. In a study of binding 17 different low molecular weight Immucillin inhibitors to the first subunit of human purine nucleoside phosphorylase (PNP) (Edwards et al. 2009), the Gibbs energy remained fairly constant at −40 to −50 kJ/mol, but the enthalpy changed from −92 to −33 kJ/mol and the entropy factor from +35 to −10 kJ/mol, values demonstrating substantial enthalpy–entropy compensation in a process that is largely enthalpy-driven (Fig. 1). Although *T*∆*S* varies by as much as 45 kJ/mol, the authors comment that the entropic term originates in protein dynamic structural changes rather than conformationally flexible states of the inhibitors or the order parameters for bound water.

**Fig. 1:**
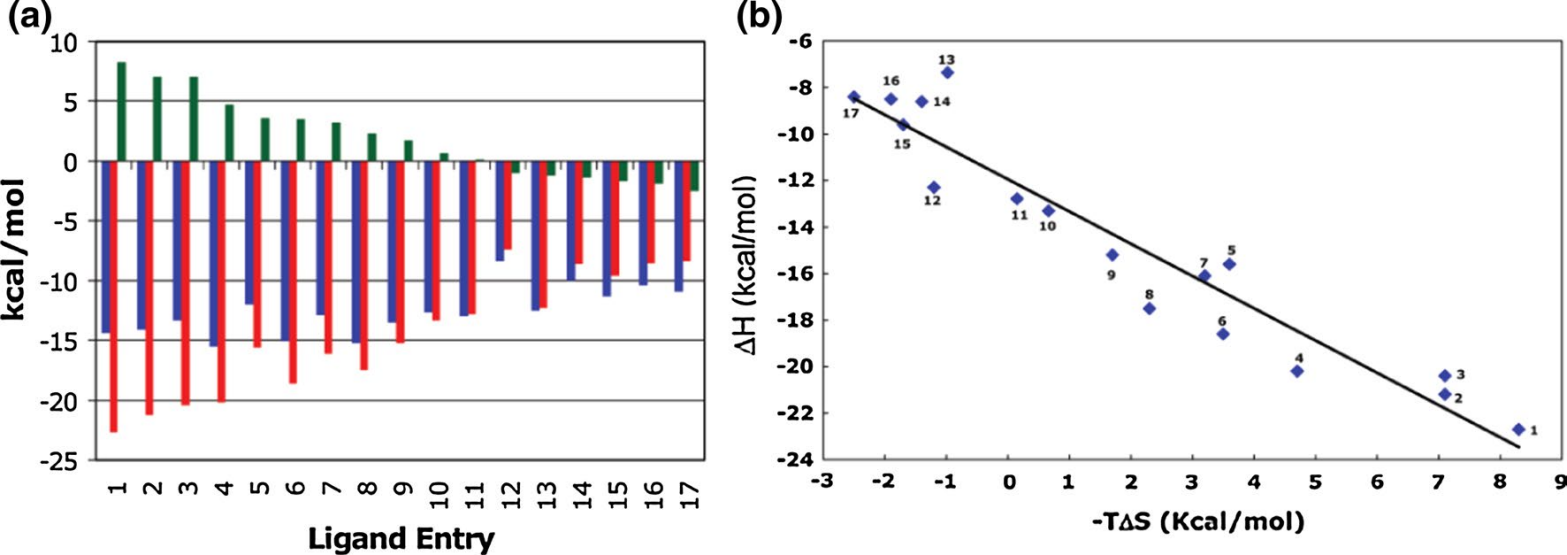
**a** Thermodynamic signatures of a variety of Immucillins binding to the first subunit of human PNP at 300 K: Δ*G* (*blue*), Δ*H* (*red*), and −*T*Δ*S* (*green*), exhibiting EEC compensation. **b** Plot of the data showing that as the enthalpy, Δ*H*, becomes more positive by about 60 kJ/mol, the entropy factor −*T*Δ*S* compensates by becoming more negative by about 45 kJ/mol. From Edwards et al. (2009)
2. A meta-analysis of ligand (effector) binding to riboswitches (Zhang et al. 2014) showed a striking level of enthalpy-entropy compensation (Fig. 2). This covered a range of ~200 kJ/mol from the entropically driven glycine riboswitch to the strongly enthalpy driven cyclic-di-GMP riboswitch. The small glycine molecule binds to a pre-existing site but the much larger cyclic-di-GMP remodels loops in the folded RNA. Riboswitches have evolved a combination of long-range tertiary interactions, conformational selection and induced fit to bind ligands of variable structure, but despite the considerable diversity in the structural reorganization of riboswitches on binding effector molecules, EEC is observed. The authors are undecided as to whether the compensation observed is conformationally based or should be attributed to changes in solvation.

**Fig. 2:**
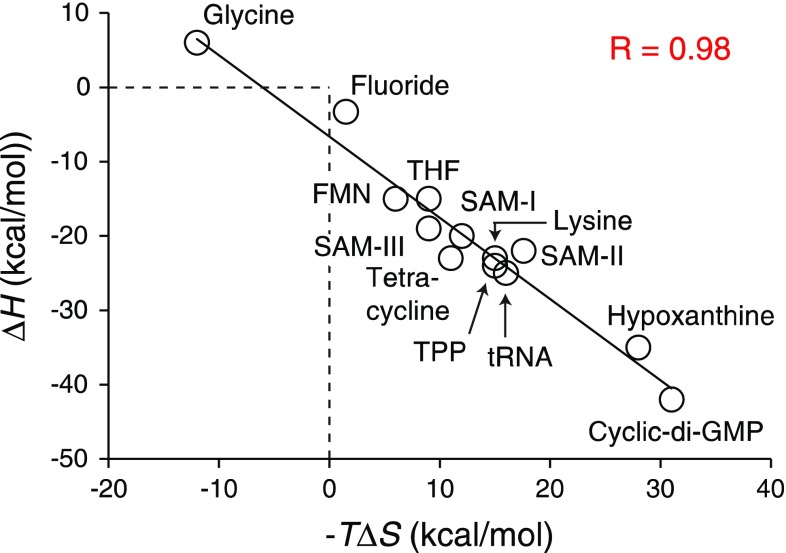
The enthalpy and entropy factors of riboswitches activated by a variety of effectors at temperatures between 288 and 310 K. From Zhang et al. (2014)
3. The participation of tightly bound water molecules in ligand–protein interactions has been investigated in some detail for the binding of phospho-tyrosine containing peptides to the SH2 domain, an interaction involving ‘indirect readout’ of the SH2 surface by bound waters, as shown by crystallography for the pYEEI peptide (Waksman et al. 1993). Using a panel of five closely related peptides, the number of waters tightly bound to the several complexes was monitored (in the gas phase) using ESI–MS and shown to vary from 3 to 5. Calorimetrically, the maximum extent of EEC amounted to ~10 kJ/mol but no obvious link was evident between the level of compensation and the number of bound waters (Chung et al. 1998). However, bearing in mind that the association of a single water molecule having the thermodynamic characteristics of ice would result in heat release of about 6 kJ/mol, such compensation could result from the binding/release of very few waters.
4. The binding to human carbonic anhydrase (HCA) of a series of benzothiazole sulfonamide ligands having different patterns of fluorination in the benzo-group was studied in Breiten et al. (2013). Despite the structural changes in the ligands being quite small and HCA being conformationally rigid upon binding of arylsulfonamide ligands, enthalpy-entropy compensation was observed over about 25 kJ/mol for an essentially constant Gibbs energy. The authors concluded that the most plausible candidate for the source of these compensating changes in Δ*H* and *T*Δ*S* is the network of hydrogen-bonded waters in the active site and surrounding the ligands, both in solution and in the protein–ligand complex. Such a process of ‘solvent reorganization’ is discussed in detail in Grunwld and Steel (1995).

## Interactions between macromolecules

**Fig. 3:**
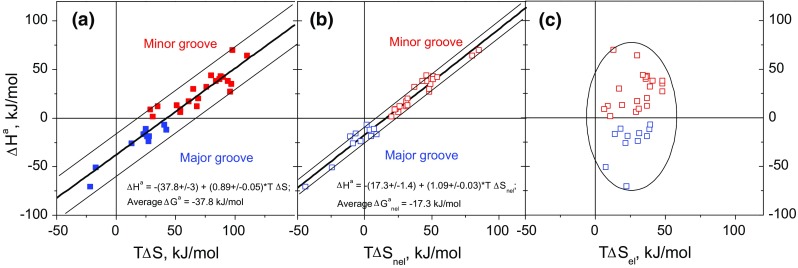
Energetics of DBD binding to target DNAs: dependence of the entropy factor, TΔS, on the enthalpy, Δ*H*. Minor groove binders, *red symbols*; major groove binders, *blue symbols*. **a** Total entropy factors, **b** non-electrostatic entropy factors, **c** electrostatic entropy factors. Data, all at 20 °C, taken from Table 1 in the Supplementary data to Privalov et al. (2007) and from Dragan et al. (2006, 2004a, b, 2003a, b) and Hargreaves et al. (2005)

In interactions between two macromolecules—for which the interaction interface may be quite large—evidence for the participation of water can be particularly strong. The first clear demonstration that EEC is a feature of protein–DNA interactions was made by Jen-Jacobson et al. (2000) who collated enthalpy and entropy data obtained at 25 °C for ten DNA binding domains (DBDs) interacting with the major groove. Subsequently, we expanded calorimetric studies to the binding of 27 DBDs to target DNA sequences, both in the major (blue) and in the minor (red) groove (see Fig. 3). In cases for which the protein component was incompletely folded in free solution, corrections were made for enthalpies of refolding and in some cases correction was also made if the temperature of measurement was such that the complex formed was slightly unfolded; see Ref. (Privalov et al. 2007) for details. It follows that reported enthalpies represent the binding of fully folded proteins to dsDNA.

Panel a plots the measured enthalpies of binding, Δ*H*, against the total entropy factors, *T*Δ*S*. The approximate linearity of the plot over a range of about 130 kJ/mol is a visual indication of compensation and the ordinate value of −37.8 kJ/mol at zero *T*Δ*S* represents the average—and fairly constant—Gibbs free energy of binding. By monitoring the salt dependence of the binding constant, a separation can be made of the non-electrostatic and electrostatic components of the total Gibbs energies (Privalov et al. 2011). The electrostatic component results from the formation of ionic contacts between basic amino acids and DNA phosphates, which release counter-ions generating an entropy factor increase of about 4 kJ/mol per salt link in a non-enthalpic process. The magnitudes of this effect (*T*Δ*S*_el_ ranging from about 8 to 48 kJ/mol, i.e., 2–12 ionic bonds) is shown in panel c, from which it is seen there is no correlation between the number of salt links in a complex and the enthalpy of the binding process. Subtracting this electrostatic component from the total entropy gives the non-electrostatic entropy factor (*T*Δ*S*_nel_), which is plotted against Δ*H* in panel b: the ordinate value of −17.3 kJ/mol at zero *T*Δ*S* represents the average non-electrostatic component of the binding free energy—which is much more constant than the total free energy. The much-improved linearity in panel b shows that the scatter in panel a is largely due to variation in the number of ionic links formed in the different complexes. The non-electrostatic entropy therefore compensates the enthalpy of binding with significant precision (the slope is very close to unity) over a range of ~130 kJ/mol, i.e., enthalpy–entropy compensation is an entirely non-electrostatic phenomenon.

What then is the basis for the very wide range of Δ*H*/*T*Δ*S*_nel_ values observed? The data were obtained for a set of complexes in which the structural basis for the protein/DNA interaction varies considerably. At one end of the spectrum is the insertion of single α-helices into the major groove (GCN4/AP-1 scissors grip, ∆*H* ~ −71 kJ/mol), with essentially no DNA distortion, to HMG box binding to the minor groove (HMGD-74, ∆*H* ~ +70 kJ/mol), involving wedge insertion and considerable DNA bending, at the other end. So is it the structural mechanism of interaction that determines the changing magnitudes of Δ*H*/*T*Δ*S*_nel_? The size and nature of the interface formed in DBD–DNA complexes is typically expressed in the magnitude of ∆ASA, the reduction in accessible surface area on forming the complex. It is striking that for most cases in Fig. 3 this amounts to roughly 1000 Å^2^, subdivided approximately equally into polar and non-polar components. As Δ*H* depends on the number of van der Waals contacts/H-bonds formed and the conformational TΔ*S*_nel_ is assumed to depend on the reduction in interfacial mobilities, these quantities should be roughly the same for all the complexes, not spread over a large range. It follows that the substantial changes in Δ*H*/*T*Δ*S*_nel_ do not reflect variations in the interfaces that are formed. The changes can only be the consequence of an external factor: in particular, changing hydration.

The importance of hydration can be seen from simple inspection of panel b: for example, the DNA binding of two HMG box factors (HMGD-100 and HMGD-74, two red circles, top right) is accompanied by an entropy factor increase of about 80 kJ/mol. On the basis of the crystallographic and NMR structures of these complexes and the free proteins (Dow et al. 2000; Murphy et al. 1999) this very large entropy increase is unlikely to result from large conformational changes—the complexes are not highly disordered relative to the separated components—it can only result from water release (Dragan et al. 2003b). It follows that the plot in panel b of Fig. 3 can be seen as a graph of varying hydration. If one makes the working assumption that tightly bound water has the thermodynamic properties of ice (the melting entropy factor of which, *T*∆*S*_nel_, is ~6.5 kJ/mol at 20 °C), then an increase in the entropy factor of 83 kJ/mol (HMGD-74, top right, red circle) represents the release of ~13 water molecules. Correspondingly, a reduction in the entropy factor of 44 kJ/mol (GCN4 with AP-1 DNA, bottom left, blue circle) represents the binding of ~7 water molecules. Since the binding/release of ice-like bound water at a temperatures of 293 K involves a Gibbs energy change close to zero, the corresponding enthalpy changes will be compensatory.

As the large fluctuations in the enthalpies and entropies of binding are largely a consequence of changing solvation, the average non-electrostatic Gibbs energy of −17.3 kJ/mol (Panel b) represents the specific part of the interaction and the addition or subtraction of water molecules has only a slight effect on the Gibbs energy. To this, in real complexes, is added a variable number of ionic bonds: these enhance the affinity considerably, and in proportion to their number, by increasing the entropy of the interactions—with no effect on the enthalpy. This suggests that a good way of improving the affinity of lead compounds would be to engineer the presence of additional ionic links.

## The GCN4–DNA (bZIP) complex

**Fig. 4:**
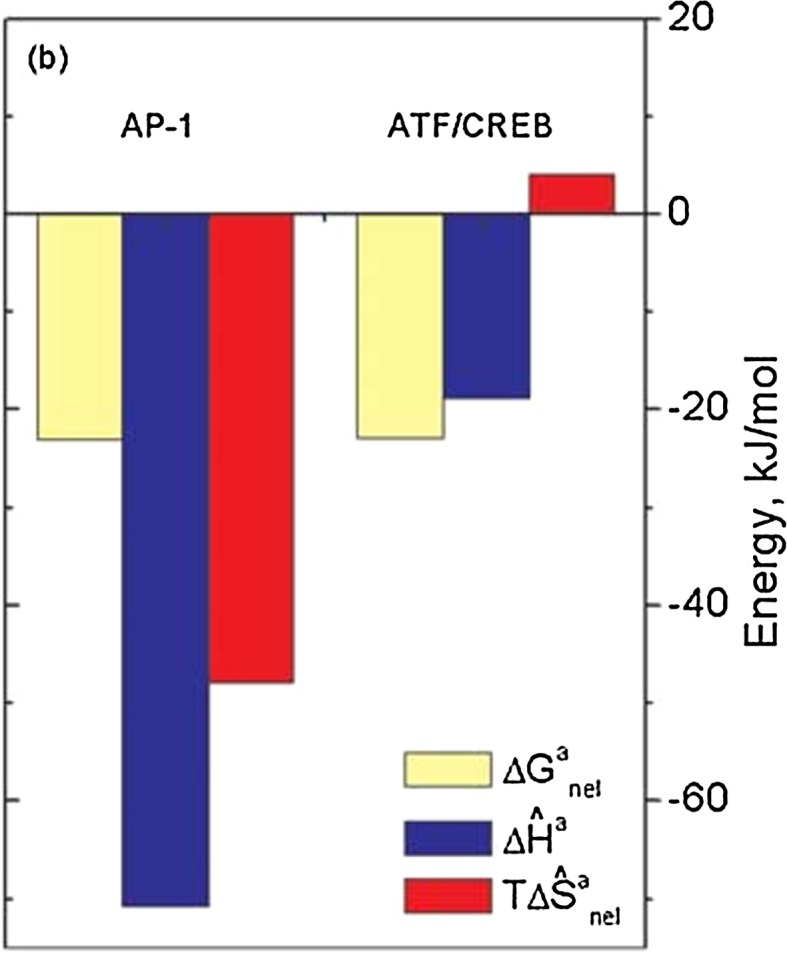
The enthalpic (Δ*H* ^a^, *blue*) and non-electrostatic entropy factor ($T\Delta S_{{_{\text{nel}} }}^{\text{a}} ,$ *red*) contributions to the non-electrostatic Gibbs energy of association ($\Delta G_{\text{nel}}^{\text{a}} ,$ *cream*) of the fully folded and crosslinked GCN4 bZIP homodimer with the AP-1 and ATF/CREB DNA target sites at 20 °C. From Dragan et al. (2004b)

A particularly revealing example of enthalpy–entropy compensation from the data set of Fig. 3 is the yeast bZIP DBD from GCN4, binding as a crosslinked homodimer in a scissors grip to two closely related target DNA sequences, AP-1 and ATF/CREB, differing in length and sequence by just one base pair (Dragan et al. 2004b). It is particularly important to recall for these two cases that correction has been made for the refolding of the basic domains into α-helices and thus the binding data represent association of fully folded GCN4 dimer to the target DNAs. The electrostatic contribution to binding is ∆*G*_nel_ = −23 kJ/mol, corresponding to formation of six ionic links, and is, as expected, identical for the two target sequences. In contrast, the non-electrostatic component varies dramatically with the target sequence: interaction with AP-1 DNA is characterized by an unusually large negative enthalpy and non-electrostatic entropy as compared to the ATF/CREB target, for which the entropy factor is even slightly positive (see Fig. 4).

The enthalpies of binding are −71 and −19 kJ/mol, respectively (dark blue in Fig. 4), while the non-electrostatic entropy factors are −44 and +4 kJ/mol, respectively (red in Fig. 4)—effectively compensating. There is thus a difference in the non-electrostatic entropy factors of 48 kJ/mol between the two complexes and to decide whether this is due to conformational or hydration differences requires separation of these two components. Estimation of the conformational component of globular protein folding/unfolding has been made by taking experimental entropies of unfolding and subtracting from them the measured entropies of solvation of the component amino acids (Makhatadze and Privalov 1995): these average to Δ*S* ^conf^ ~ 50 J/K per mol of amino acid. Theoretical estimates of the conformational entropy cost of protein folding (D’Aquino et al. 1996; Baldwin 2007; Doig and Sternberg 1995) lead to values of Δ*S* ^conf^ ~ 28 J/K per mol of amino acid. An average of these two figures (Δ*S* ^conf^ ~ 40 J/K per mol of amino acid) thus represents the best present estimate, so at 20 °C the conformational entropy factor of protein unfolding, TΔS, is about 12 kJ per mol of amino acid.

It follows that if the 48 kJ/mol difference in the entropy factors between the two GCN4 complexes has a solely conformational explanation, the structural changes would be equivalent to the folding (immobilization) of four amino acids. However, the crystal structures of the complexes with the two target DNAs are very similar (Ellenberger et al. 1992; Keller et al. 1995): the two α-helix backbones overlap with a RMSD of only 1.3 Å. The only notable difference between the two structures is the changed interaction of a single conserved Arg sidechain (R243) that binds close to the center of the target DNA. These conformational differences are very small compared to that for the unfolding four amino acids.

Equally, the concomitant enthalpy difference of 52 kJ/mol is too large to be a consequence of differences in DNA–protein contacts, bearing in mind that the enthalpy of forming a hydrogen bond is estimated at only 3 kJ/mol (Taylor et al. 1999). The most reasonable explanation for these very large discrepancies in the entropies and enthalpies of forming the two very similar GCN4 complexes is differences in the number of incorporated ordered water molecules. If, as above, we approximate the entropy loss upon immobilization of a water molecule as similar to that of freezing water, it follows that the AP-1 complex has seven or eight more incorporated water molecules than the ATF/CREB complex.

## A protein–protein interaction (calmodulin)

**Fig. 5:**
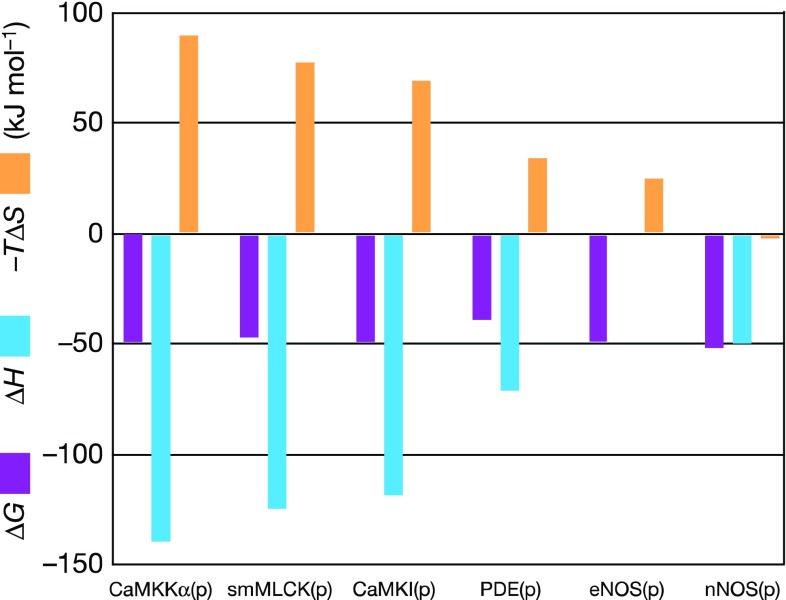
The Gibbs free energy (Δ*G*, *purple*), enthalpy (Δ*H*, *blue*) and entropy factor (−*T*Δ*S*, *yellow*) for the formation of six calcium-saturated CaM–peptide complexes at 35 °C, determined by isothermal titration calorimetry. From Frederick et al. (2007)

An example of a protein–protein interaction in which modifications to one of the components results in large variations in the enthalpy and entropy of binding, despite the overall Gibbs energy remaining little changed, concerns the binding to calmodulin (CaM) of a set of peptides (19–25 amino acids) derived from target domain proteins (Frederick et al. 2007), see Fig. 5.

Although the Gibbs energies of binding (∆*G*, purple) are all close to −50 kJ/mol, the calorimetrically measured entropy factors (*T*∆*S*, yellow) range from −90 to +2 kJ/mol: a variation of 92 kJ/mol. This is twice the difference observed for the two GCN4 targets. The structures of the CaM–peptide complexes are closely related, though the peptides themselves differ in sequence. NMR relaxation methods were used (Frederick et al. 2007) to monitor the mobility of calmodulin methyl groups in the complexes as a proxy for local order/disorder, i.e., for the conformational entropy change on binding. A good correlation was observed between entropies derived from calorimetric data and the methyl mobilities, suggesting that changes in the conformational entropy of calmodulin on forming its peptide complexes are a major contributor to the binding entropy. In fact, it was assumed (Marlow et al. 2010) that all of the calorimetrically measured entropy changes were of conformational origin, thereby allowing a calibration of the methyl group mobilities in entropic terms. However, since changes in the enthalpies of binding are also very large, alterations in the levels of hydration are likely contributors to the calorimetrically measured values. The authors indeed comment: ‘the linearity of the correlation implies that either the change in the conformational entropy of calmodulin on binding a target domain is a major contribution to the binding entropy or that the various sources of entropy change in concert’ (Frederick et al. 2007)—so could it be that levels of hydration change in concert with the conformational entropy of calmodulin? Consideration of the following protein–DNA interaction case makes this a very plausible scenario.

## The catabolite activator protein (CAP)–DNA complex

**Fig. 6:**
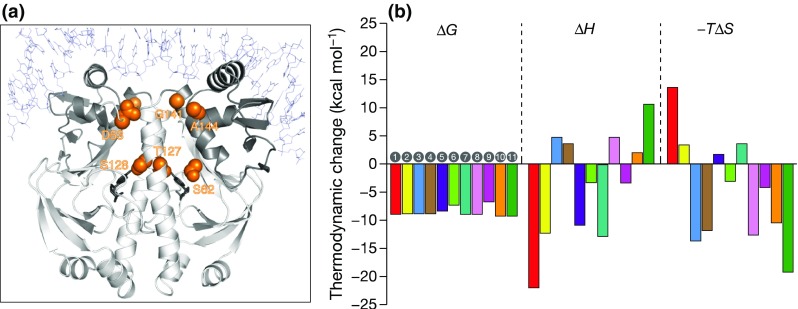
**a** The CAP homodimer, ligated with two molecules of cAMP [CAP-cAMP2] bound to a 22-bp DNA target duplex. The residues subject to mutation are highlighted in *orange*: S62, T127/S128, A144, G141 and D53. They do not participate in the interaction with DNA. **b** Calorimetrically determined components (Δ*G*, Δ*H*, and −*T*Δ*S*) for the binding of the CAP variants to DNA at 305 K. From Tzeng and Kalodimos (2012)

A protein–DNA interaction in which very large entropy differences have been interpreted solely in conformational terms without considering major solvation changes, concerns the binding of a substantial set of single amino acid allosteric mutants of the catabolite activator protein (CAP) homodimer to a unique target DNA (Tzeng and Kalodimos 2012), see Fig. 6a.

Again it was observed (Fig. 6b) that although the Gibbs energies of binding varied only modestly (between −31 and −37 kJ/mol), the enthalpy (∆*H*) and entropy factor (−*T*∆*S*) components changed dramatically: the binding enthalpy varied from −92 to +44 kJ/mol and, correspondingly, the entropy factor from –54 to +79 kJ/mol, a total of 133 kJ/mol, almost three times that seen in the GCN4–DNA interaction. This system therefore exhibits EEC over as broad a range as the DBD–DNA interactions shown in Fig. 3. The five residues subjected to mutation were all in the body of the CAP protein (Fig. 6a), the majority not too distant from the protein–protein dimer interface and not at the protein–DNA interface.

**Fig. 7:**
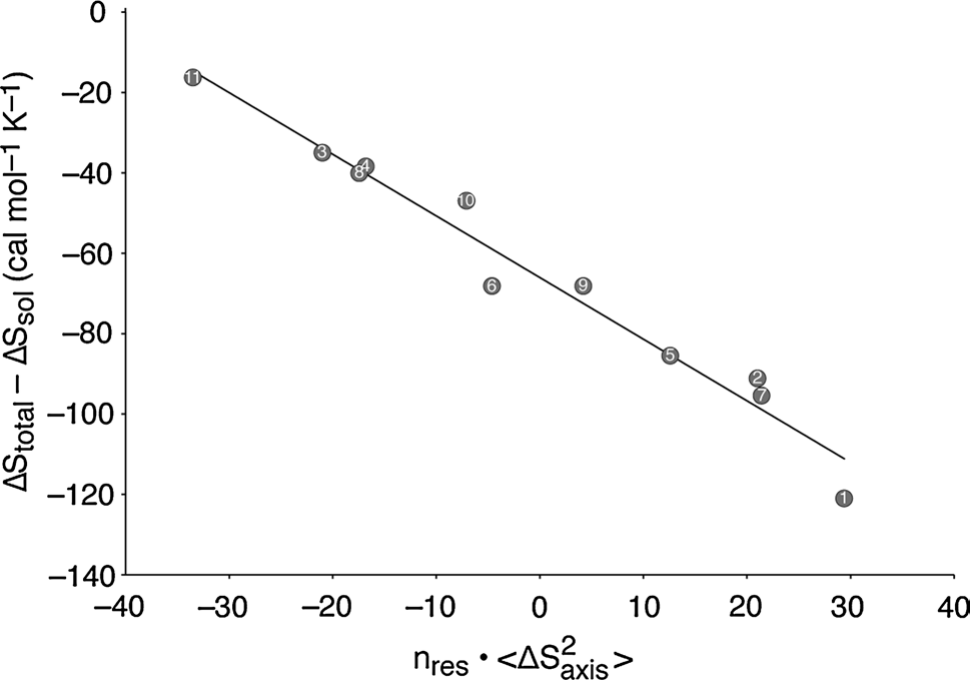
Correlation between the total calorimetric entropy of binding (Δ*S*_total_), from which is subtracted (Δ*S*_sol_) the calculated entropy of solvent release from the CAP-DNA interface—with the average change in the methyl mobility factor, $S_{\text{axis}}^{2}$, of CAP upon DNA binding ($\Delta S_{\text{axis}}^{ 2}$). The samples are numbered as in Fig. 6b: No. 1 being WT CAP with bound cAMP_2_ and No. 11 being the A144T mutant with bound cGMP_2_. *n*_res_ = 418 is the number of residues in dimeric CAP. From Tzeng and Kalodimos (2012)

NMR relaxation methods were used to monitor the mobility of methyl groups in the CAP protein as a proxy for the degree of order present—as in the CaM-peptide case. A good linear correlation was observed between these averaged mobilities and the calorimetrically derived entropies of binding, i.e., changes in protein order correlate with changes in binding entropies, see Fig. 7.

NMR studies of the variant complexes indicated an unchanging DNA interface, so these ‘alternative thermodynamic strategies’ adopted by the variant CAP dimers cannot be a consequence of alterations in CAP-DNA contacts, nor result from differences in the amounts of solvent released on forming the protein–DNA interface. The large calorimetric entropy variations were therefore ascribed solely to changes in the conformational entropy of the CAP protein mutants and it was concluded that these drive the energetics of binding. This, of course, raises the question as to the source of the large concomitant changes in the binding enthalpy, 136 kJ/mol. Noting the strong enthalpy–entropy compensation and the unchanging CAP–DNA interface, structural changes within the CAP protein, e.g., at the dimer interface, would have to be responsible for the enthalpy change of 136 kJ/mol and for 133 kJ/mol in *T*∆*S* if changes in hydration are excluded. For a 133 kJ/mol change in *T*∆*S* to be all conformational, then on the basis of the conformational entropies discussed above for the GCN4 complexes, it would be equivalent to the unfolding of 133/12 = ~11 amino acids. This seems much more than expected from the set of single-residue mutants studied. We conclude that with such large changes of both enthalpy and entropy to be explained, substantial concomitant changes in hydration must accompany the conformational changes.

## Conclusions

In the macromolecular examples summarized above, the Gibbs energies of binding alter little and the large changes in enthalpy and entropy are too great to be a consequence of only conformational changes. They must therefore result, at least partially, from variations in the amounts of water immobilized or released on forming the complexes. For both the CaM/peptide and CAP systems, the correlation between the calorimetric entropies and the local mobilities is very clear, but it does not follow that conformational changes are the sole contributor to the entropy: hydration changes must also be involved. It follows that there must be a structural link between the amount of bound water and the protein flexibility. The most rigid examples in these two systems [wt CAP/DNA—Sample 1 in Figs. 6b, 7—and CaM:CaMKKα(p) in Fig. 5] exhibit the greatest entropy decrease on forming the complex and, correspondingly, the most flexible examples [CAP-A144T-cGMP_2_/DNA—Sample 11 in Figs. 6b, 7—and CaM:nNOS(p) in Fig. 5] exhibit the most positive entropy changes. It follows that the more rigid is the complex formed, the more water is immobilized and increasing flexibility results in immobilization of reducing numbers of water molecules. It therefore seems that the most flexible protein systems fix the least water and the most rigid fix the most.

The relationship between the flexibility of a protein chain and the nature of its bound water has been investigated in detail for hen egg white lysozyme (King et al. 2012). Using site-specific probes to monitor the state of solvating water molecules, it was shown that whereas water bound to a rigid hydrophobic patch on the protein surface was dynamically constrained, the water surrounding a flexible and looser part of the fold preserved the dynamics of bulk water. Deposition of water onto rigid hydrophobic protein surfaces will result in an entropy reduction, but not deposition where the protein structure is flexible: this difference would result in the observed correlation between conformational flexibility in the CAP variants and the entropy of their DNA binding (Fig. 7). It is noteworthy that as CAP flexibility was monitored through methyl groups, it represents the more hydrophobic parts of the protein. The situation can be expressed as follows: the introduction of mutations into wt CAP makes the structure looser and flexible, resulting in an increasing loss of dynamically constrained water from the dimer. The consequence is an increasingly positive contribution to the calorimetric entropy (and enthalpy) of binding which correlates with the NMR manifestations of protein flexibility—as observed by Tzeng and Kalodimos (Fig. 7). We conclude that the increasingly positive calorimetric entropies have a large contribution from the loss of bound water, in addition to conformationally based increasing flexibility.

Finally, it is important to re-emphasize that the large entropies and enthalpies associated with the binding/release of dynamically constrained water from macromolecular systems are intrinsically compensatory: if such water has an ice-like structure and the temperature is 273 K, then there would be absolutely no consequent change in the Gibbs energy. This represents the main energetic basis of enthalpy–entropy compensation.
